# Allopurinol reduces oxidative stress and activates Nrf2/p62 to attenuate diabetic cardiomyopathy in rats

**DOI:** 10.1111/jcmm.14870

**Published:** 2019-12-19

**Authors:** Jierong Luo, Dan Yan, Sisi Li, Shiming Liu, Fei Zeng, Chi Wai Cheung, Hong Liu, Michael G. Irwin, Huansen Huang, Zhengyuan Xia

**Affiliations:** ^1^ Department of Anesthesiology The Second Affiliated Hospital Guangzhou Medical University Guangzhou China; ^2^ Department of Anesthesiology The University of Hong Kong Hong Kong China; ^3^ Department of Anesthesiology and Pain Medicine University of California Davis Health System Sacramento CA USA

**Keywords:** allopurinol, autophagy, diabetic cardiomyopathy, Nrf2, oxidative stress

## Abstract

Allopurinol (ALP) attenuates oxidative stress and diabetic cardiomyopathy (DCM), but the mechanism is unclear. Activation of nuclear factor erythroid 2‐related factor 2 (Nrf2) following the disassociation with its repressor Keap1 under oxidative stress can maintain inner redox homeostasis and attenuate DCM with concomitant attenuation of autophagy. We postulated that ALP treatment may activate Nrf2 to mitigate autophagy over‐activation and consequently attenuate DCM. Streptozotocin‐induced type 1 diabetic rats were untreated or treated with ALP (100 mg/kg/d) for 4 weeks and terminated after heart function measurements by echocardiography and pressure‐volume conductance system. Cardiomyocyte H9C2 cells infected with Nrf2 siRNA or not were incubated with high glucose (HG, 25 mmol/L) concomitantly with ALP treatment. Cell viability, lactate dehydrogenase, 15‐F2t‐Isoprostane and superoxide dismutase (SOD) were measured with colorimetric enzyme‐linked immunosorbent assays. ROS, apoptosis, was assessed by dihydroethidium staining and TUNEL, respectively. The Western blot and qRT‐PCR were used to assess protein and mRNA variations. Diabetic rats showed significant reductions in heart rate (HR), left ventricular eject fraction (LVEF), stroke work (SW) and cardiac output (CO), left ventricular end‐systolic volume (LVVs) as compared to non‐diabetic control and ALP improved or normalized HR, LVEF, SW, CO and LVVs in diabetic rats (all *P* < .05). Hearts of diabetic rats displayed excessive oxidative stress manifested as increased levels of 15‐F2t‐Isoprostane and superoxide anion production, increased apoptotic cell death and cardiomyocytes autophagy that were concomitant with reduced expressions of Nrf2, heme oxygenase‐1 (HO‐1) and Keap1. ALP reverted all the above‐mentioned diabetes‐induced biochemical changes except that it did not affect the levels of Keap1. In vitro, ALP increased Nrf2 and reduced the hyperglycaemia‐induced increases of H9C2 cardiomyocyte hypertrophy, oxidative stress, apoptosis and autophagy, and enhanced cellular viability. Nrf2 gene silence cancelled these protective effects of ALP in H9C2 cells. Activation of Nrf2 subsequent to the suppression of Keap1 and the mitigation of autophagy over‐activation may represent major mechanisms whereby ALP attenuates DCM.

## 1 INTRODUCTION

Diabetic cardiomyopathy (DCM), a common complication of diabetes mellitus, is the greatest mortality risk for patients with diabetes.^1^ However, DCM is largely unrecognized in asymptomatic diabetic stage despite of the pathological progression.^2^ Hyperglycaemia induced excessive production of reactive oxygen species (ROS), and the subsequent oxidative stress play critical roles in this pathology.^3^, ^4^ Cardiomyocyte hypertrophy, apoptosis and fibrosis have long been considered as three major features of DCM which eventually progress to heart failure. Antioxidant treatments as therapeutic strategies against DCM have been multi‐dimensionally studied both clinically and experimentally.^5^ However, convincing benefits of antioxidant therapy have not been seen in patients with diabetes because of limited understanding of the mechanism.

Nuclear factor erythroid 2‐related factor 2 (Nrf2), a transcription factor that can be activated by oxidative stress, regulates antioxidant response elements (ARE) that control the basal and inducible expression of antioxidant genes in response to xenobiotics, antioxidants, heavy metals and UV light.^6^, ^7^ In the presence of oxidative stress, Nrf2 confers protective effects via translocation into the nuclear and subsequently activate ARE to induce antioxidant genes, such as NQO1, GCLC, heme oxygenase‐1 (HO‐1) and SQSTM1.^8^, ^9^ We recently showed that cardiac Nrf2 was reduced in streptozotocin (STZ)‐induced diabetic rats and strategies that enhanced cardiac Nrf2 could activate myocardial HO‐1 and attenuate cardiac hypertrophy and cardiac dysfunction in diabetic rats.^10^ Also, recent studies suggest that targeting Nrf2 may represent a mechanism by which treatments that can attenuate DCM.^11^, ^12^ However, the potential impact of allopurinol (ALP) on cardiac Nrf2 expression in diabetes and its underlying mechanism of action have not been explored.

Nrf2 is activated following the disassociation with its repressor Kelch‐like epichlorohydrin‐associated protein 1 (Keap1), a cytosolic repressor protein of Nrf2. Keap1 binds and retains Nrf2 in the cytoplasm, preventing Nrf2's nuclear translocation.^13^, ^14^ Interestingly, among the downstream Nrf2‐driven genes, p62, which is encoded by SQSTM1,^15^ serves as a specific autophagy receptor for Keap1.^16^ The autophagic cargo adaptor p62 conjugates with Keap1, embedding it together with aggregates of other ubiquitinated proteins for degradation, which liberates and activates Nrf2.^15^ Autophagy is a dynamic process that is tightly regulated by cellular metabolic balance, and functional autophagy is essential for cell survival. Given the coexistence of elevated oxidative stress, increased protein and lipid oxidation, and impaired cellular energy balance in diabetes, functional autophagy may be of greater importance in maintaining cardiac cell integrity in diabetes.^17^ Based on aforementioned findings, it is apparent that there exits an interconnected loop linking Nrf2, Keap1 and p62 in vivo, which functions collectively to maintain homeostasis during oxidative stress or other injury stresses. However, whether or not the development of DCM involves this loop and how this loop varies in diabetes mellitus and in particular the diabetic myocardium is unclear.

Allopurinol, a competitive inhibitor of xanthine oxidase enzyme, has dose‐dependent free radical scavenging property.^18^ It attenuates the diabetes‐induced increase of myocardial xanthine oxidase activity,^19^, ^20^ reduces ROS production and improves diabetes‐induced cardiac dysfunction.^21^ Our previous studies demonstrated that ALP could ameliorate diabetic myocardial ischaemia/reperfusion (MI/R) injury by lowering ROS production.^22^ Meanwhile, Demirel et al^23^ showed that ALP could alleviate acute liver failure via regulating cellular redox‐sensitive transcription factor Nrf2, indicating that ALP conferred protection through interacting with Nrf2 signalling pathway. A recent study showed that myocardial autophagy participated in the modulation of the development of DCM.^24^ Thus, it is possible that ALP may ameliorate DCM via regulating Nrf2 signalling pathway.

We suggested that the hyperglycaemia‐induced oxidative stress and the over‐activation of myocardial autophagy are key mechanisms that cause the imbalance of Nrf2‐Keap1‐p62 and the development of DCM. ALP treatment may activate Nrf2 to mitigate autophagy over‐activation and consequently attenuate DCM. This hypothesis was tested both in type 1 diabetic rats and in H9C2 cardiomyocytes exposed to high glucose.

## 2 MATERIALS AND METHODS

### 2.1 Animals and induction of diabetes

Following the approval by institutional Animal Research Committee, specific pathogen‐free (SPF) male Sprague Dawley rats weighing rats (250 ± 10 g, 6‐8 weeks) were obtained and housed for 7 days after arrival in the animal facility before performing the experiments. The rats received standard care which meets *the International Guiding Principles for Biomedical Research Involving Animals*, as issued by the Council for the International Organizations of Medical Sciences. Type 1 diabetes was induced as described.^25^ In brief, streptozotocin (STZ) was injected via tail vein at a dose of 65 mg/kg body weight (Sigma‐Aldrich) in 0.1 mol/L citrate buffer (pH 4.5) or citrate buffer alone as control under anaesthesia. One week after STZ injection, rats exhibiting hyperglycaemia (blood glucose 16.7 mmol/L or higher) were considered type 1 diabetic and subjected to subsequent experiments. Consistent with our previous study which showed that STZ induced significant reduction in insulin and increase in plasma glucose and that antioxidant treatment did not have significant impact on plasma levels of insulin and glucose,^26^ our preliminary experiment showed STZ significantly decreased plasma insulin level in diabetic rats compared with controls (data not shown) with concomitant increase in plasma glucose. Therefore, in the current study, hyperglycaemia‐mediated cardiac complications and mechanism were our major targets; therefore, we did not specifically measure insulin in diabetic rats but confirmed the successful establishment of diabetic model by using plasma glucose as a major indicator.

### 2.2 Experimental protocol

Rats were randomly divided into three groups (n = 6 per group): control (C), diabetes (D) and diabetes treated with ALP (DA). ALP (Sigma‐Aldrich) were dissolved in drinking water for 4 weeks' duration of treatment starting 1 week after induction of diabetes. We used a dose of ALP at 100 mg/kg/d, which has been demonstrated to attenuate heart injury from ischaemia/reperfusion in our previous study.^22^ The daily dose of ALP (100 mg/kg) was dissolved into 2/3 volume of the average daily amount of drinking water that was estimated based on preliminary study and previous studies to ensure that the total amount of ALP was taken by the rats before additional amount of water was supplied. Meanwhile, the plasma glucose, body weight, water intake and food consumption of rats were routinely monitored. At the end of the experiments, cardiac function was determined by the echocardiography (4 weeks after diabetes induction) and pressure‐volume conductance catheter system (5 weeks after diabetes induction), respectively, before the rats was terminated. After the completion of experiments, rats were then deeply anaesthetized with sodium pentobarbital (65 mg/kg), and hearts were rapidly excised and frozen in liquid nitrogen for later analysis.

### 2.3 Measurement of left ventricular function

#### 2.3.1 Echocardiography

Rats from each group were anaesthetized by inhalation of 3% isoflurane in pure O_2_, which was continuously maintained till the end of the echocardiography.^27^ Parasternal Short Axis View (PSAX) was selected, and M‐mode echocardiography was performed by using a 13‐24 MHz liner array transducer system (Vevo 770TM High Resolution Imaging System; VisualSonics). The short axis view was acquired at the level of papillary muscles. HR (heart rate) and left ventricle (LV) structural changes, including LVIDs (LV internal diameter in systole), LVIDd (LV internal diameter in diastole), LVAWd (LV end‐diastolic anterior wall diameter), LVAWs (LV end‐systolic anterior wall diameter), LVPWs (LV posterior wall thickness in systole) and LVPWd (LV posterior wall thickness in diastole), were measured through the M‐mode echocardiography images. LVM (LV mass) was calculated using the following equation:

$$\text{LVM}\;(g)=1.04\times [{\left(\text{LVIDd}+\text{IVSd}+\text{LVPWd}\right)}^{3}-{\text{LVIDd}}^{3}]\times 0.8+0.6.$$

Meanwhile, LV contractile function parameters FS (fractional shortening), EF (ejection fraction) and SV (stroke volume) were calculated using LVIDs, LVIDd, LVVd (LV end‐diastolic volume) and LVVs (LV end‐systolic volume) as described.^28^ Three representative cardiac cycles were recorded and averaged for each measurement.

#### 2.3.2 Intracardiac heart function detection

The global cardiac functions were monitored by using a pressure‐volume (PV) conductance catheter (AD Instruments) placed into the left ventricle through the right carotid artery and connected to a computer equipped with an advantage PV control box software (AD Instruments) as previously described.^25^ The cardiac functional parameters were recorded, including heart rate (HR), left ventricular end‐systolic pressure (Pes, LVESP), left ventricular end‐diastolic pressure (Ped), stroke volume (SV), left ventricular ejection fraction (EF), stroke work (SW) and cardiac output (CO). The load‐independent contractility parameters including the maximal slope of systolic pressure increment (d*P*/d*t*_max_), arterial elastance (Ea = LVESP/SV), diastolic decrement (d*P*/d*t*_min_) and the relaxation time constant calculated by the Weiss method (Tau) were analysed using Labchart 8 software (AD Instruments).

### 2.4 Plasma and cardiac free 15‐F2t‐isoprostane and SOD measurement

Free 15‐F2t‐IsoP, a specific marker of oxidative stress in vivo, was measured by using an enzyme immunoassay kit (Cayman Chemical) as described.^27^ The value of free 15‐F2t‐IsoP was expressed as pg/mL in plasma and as pg/mg protein in the heart tissue. Myocardial and plasma SOD activity was detected in cardiac tissue homogenates using commercially available kits (Cayman Chemical) as described previously.^29^

### 2.5 Detection of left ventricle tissue reactive oxygen species production by in situ dihydroethidium staining

Levels of in situ $O_{2}^{-}$ production were detected by dihydroethidium (DHE; Sigma‐Aldrich) staining as described.^27^ DHE binds to DNA on oxidization by $O_{2}^{-}$ to generate fluorescent ethidium bromide, and the resulting fluorescence intensity reflects the quantity of ROS production. Frozen sections of left ventricular tissue (5 μm) were incubated with 7.5 mmol/L DHE at 37°C for 30 minutes. Fluorescence images of ethidium bromide were obtained with a fluorescence microscope (BX41 System microscope; Olympus), and the images were captured by a DP72 digital camera. The fluorescence of DHE‐labelled positive nuclei was calculated in each of five randomly selected fields and are expressed as a percentage of the DHE‐stained positive myocyte nuclei compared with control by a quantitative morphometric method.^30^

### 2.6 Apoptotic cell death detection using terminal deoxynucleotidyl transferase dUTP nick‐end labelling

Terminal deoxynucleotidyl transferase dUTP nick‐end labelling (TUNEL) reaction was performed using an in situ cell death detection kit (Roche Diagnostics GmbH) as previously described.^31^ The sections were observed in the light microscope by an investigator who was initially blinded to treatment groups, and five randomly selected fields of each slide were analysed, and the apoptotic index was calculated as a percentage of apoptotic nuclei to total nuclei.

### 2.7 Transfection and siRNA knock‐down in H9C2 cells

Embryonic rat cardiac H9C2 cells from ATCC, at the passages 5‐10, were maintained in Dulbecco's modified Eagle's medium containing 10% FBS, 100 units/mL penicillin and 100 mg/mL streptomycin in a humidified atmosphere (5% CO_2_ and 95% O_2_) at 37°C. Gene silencing was performed using Nrf2 siRNA (sc‐156128; Santa Cruz Biotechnology), control siRNA (sc‐36869; Santa Cruz Biotechnology) and lipofectamine 2000 (Invitrogen). Transfection of H9C2 cells was performed following the manufacturer's instructions. Briefly, H9C2 cells were seeded in a six well tissue culture plate and allowed to reach 30%‐50% confluence. They then were transfected with targeted Nrf2 siRNA and control siRNA. Six hours after the transfection, normal growth medium (with 5.5 mmol/L glucose) was added and the cells were incubated at 37°C in a CO_2_ incubator for 24 hours. Then, the cells were divided into five groups: normal glucose (NG, 5.5 mmol/L), high glucose (HG, 25 mmol/L), high glucose and ALP (HA, 100 μmol/L), high glucose and ALP treated with control siRNA (NC), and high glucose and ALP treated with Nrf2 siRNA (Nrf2). After the transfections, cells received 48‐hour ALP treatment in the condition of high glucose except the NG group. Ultimately, the cells were used for subsequent determinations. Besides, Bafilomycin (Baf) control by incubating the H9C2 cells with 50 nmol/L bafilomycin for 4 hours before the terminal of the experiments. Each experiment was performed independently in triplicate, and the experiment was repeated two more time.

### 2.8 Measurement of cardiomyocyte surface area

H9C2 cells were stained with phalloidin‐tetramethylrhodamine conjugate (Santa Cruz Biotechnology) as previously described.^4^ The cardiomyocyte picture was obtained with a fluorescence microscope (BX41 System microscope; Olympus), and the images were captured by a DP72 digital camera with 400 magnification. The cell surface area was determined with image analysis software ImageJ 1.48 (National Institutes of Health) and calculated as the mean of 100 cells of five randomly selected fields.

### 2.9 Measurement of cellular ROS in cultured cardiomyocytes

Superoxide generation in cultured cardiomyocytes was estimated by DHE staining as previously described.^32^ Briefly, cardiomyocytes were loaded with DHE at a concentration of 10 μmol/L for 30 minutes at 37°C. The DHE fluorescence of DHE‐labelled positive nuclei was calculated in each of five randomly selected fields and was expressed as a percentage of the DHE‐stained positive myocyte nuclei compared with control by a quantitative morphometric method.

### 2.10 Determination of cellular injury

Cell lactate dehydrogenase (LDH) content was measured with a LDH Cytotoxicity Assay Kit (Roche) as described.^33^ Cell viability was measured using the CCK‐8 kit (Solarbio). In brief, H9C2 cells were seeded at 5 × 10^3^ cells per well in 96‐well plates in triplicate. After the transfection and 48 hours treatments, 10 μL of CCK‐8 solution was added to each well. After 2 hours of incubation, absorbance was measured at 450 nm.

### 2.11 Isolation of myocardial cytosolic and nuclear fractions

Hearts from control and STZ‐treated rats were cleared of blood by washing thoroughly in Tyrode buffer and aortic and atrial sections removed from the ventricles. Ventricular tissue was freeze‐clamped in liquid nitrogen and stored until fractionated to isolate cytosolic and nuclear fractions according to the manufacturer's protocol as described in the Nuclear and Cytoplasmic Extraction Kit (Thermo).

### 2.12 Extraction of total RNA and quantitative real‐time polymerase chain reaction analysis

Cardiac total RNA was extracted from frozen heart tissues with TRIzol (TaKaRa Bio, Inc.) and reversed into cDNA with PrimeScript™ RT Master Mix. Quantitative real‐time PCR was performed with a SYBR Green PCT Master Mix (TaKaRa Bio, Inc.) on a Applied Biosystems Prism 7000 Sequence Detection System as described.^34^ Gene‐specific primers were as follows:

Rat Nrf2 forward: 5′‐GCAACTCCAGAAGGAACAGG‐3′
Reverse: 5′‐AGGCATCTTGTTTGGGAATG‐3′
Rat p62 forward: 5′‐GGAACTGATGGAGTCGGATAAC‐3′
Reverse: 5′‐GTGGATGGGTCCACTTCTTT‐3′
Rat keap1 forward: 5′‐CCTGTCTGTTGTCTCTGCTTAC‐3′
Reverse: 5′‐GAAGTTGGGTCATTGGCTTCTA‐3′
GAPDH forward: 5′‐GGGTGTGAACCACGAGAAAT‐3′
Reverse: 5′‐ACTGTGGTCATGAGCCCTTC‐3′.

The reverse concentration is 500 ng of total RNA in 10 μL reaction system. The data of qPCR were analysed via mean relative content ($2^{-\Delta \Delta C_{T}}$, $\Delta \Delta C_{T}=\left(\Delta C_{T_{\text{sample}}}-\Delta C_{T_{\text{calibrator}}}\right)$).

### 2.13 Western blot analysis

Protein samples from rat heart homogenate and H9C2 cells were resolved by 7.5%‐12.5% SDS‐PAGE and equal protein amount (50 µg) subsequently transferred to polyvinylidene nitrocellulose membranes and processed as described.^35^ The primary antibodies against Keap1 (1:1000, 8047, CST), p62 (1:1000, 5114, CST), LC3 (1:1000, 4108, CST), HO‐1 (1:800, 43966, CST), cleaved caspase 3 (1:1000, 9964, CST), Bax (1:1000, 2772, CST), GAPDH (1:2000, 5174, CST) and histone 3 (1:1000, 4499, CST) were purchased from Cell Signaling Technology, Nrf2 (1:1000, ab62352; Abcam) and Bcl2 (1:1000, ab32124; Abcam) antibodies were purchased from Abcam. Second antibodies included anti‐rabbit (1:3000, 14708, CST) and anti‐mouse (1:2000, 14709, CST). Protein bands were visualized by an enzymatic chemiluminescence method and quantified with ImageJ software. All the target proteins were normalized by GAPDH and calculated percentage of the control.

### 2.14 Statistical analysis

All values are presented as means ± standard error of the mean (SEM). One‐way analysis of variance (ANOVA) was used for statistical analyses (GraphPad Software, Inc) of data obtained within the same group and between groups, respectively, followed by Tukey's test for multiple comparisons of group means. *P* values less than .05 were considered to indicate statistically significant differences.

## 3 RESULTS

### 3.1 General characteristic variations at termination

As shown in Table 1, typical type 1 diabetic symptoms polydipsia, polyphagia, emaciation and hyperglycaemia were obvious in the STZ‐induced diabetic rats. The plasma glucose level, water intake and food consumption of the diabetic rats increased while their body weight decreased compared with control groups (all *P* < .05 D vs C; DA vs C). Plasma glucose was elevated in the diabetic group as compared to controls. ALP treatment reduced the water income, but had no significant impact on food consumption, body weight and plasma glucose in diabetic rats (Table 1).

**Table 1:** Effect of allopurinol on general characteristics in controls and rats with streptozotocin‐induced diabetes

Parameters	C	D	DA
Water intake (mL/kg/d)	120.6 ± 2.5	790.4 ± 11.2*	679 ± 10*, #
Food consumption (g/kg/d)	65.5 ± 4.5	180.1 ± 12.3*	179.7 ± 8.5*
Plasma glucose (mmol/L)	7.3 ± 0.3	29.5 ± 1.5*	30.3 ± 1.2*
Body weight (g)	476.2 ± 5.0	343.3 ± 4.5*	341.2 ± 6.0*
Heart weight (g)	1.15 ± 0.03	0.96 ± 0.02*	0.96 ± 0.04*
Heart/Body weight ratio (g/kg)	2.42 ± 0.07	2.81 ± 0.05*	2.82 ± 0.11*

Note

Water intake and food consumption were the average value of 4 wk. Body weight and plasma glucose were measured at termination. Control (C), diabetes (D), diabetes treated with allopurinol (DA). Data are expressed as mean ± SEM (n = 6 per group).

* *P* < .05 vs C.

^#^ *P* < .05 vs D

### 3.2 Changes of left ventricular dimensions and function and the effects of ALP on diabetic heart dysfunction

Through echocardiography, left ventricular inner diameter and thickness of myocardium were measured and the heart function was detected (Table 2), synergizing with pressure‐volume loop system (Table 3), an intracardiac high‐fidelity ventricular pressure and volume measurement. At the early diabetic status (4 weeks), echocardiography showed inconspicuous cardiac functional damage, specifically demonstrated as reduced HR, LVEF, stroke work and cardiac output. These heart functional impairments are mainly due to the decline of cardiac systolic function, indicated by the reduction in end‐systolic pressure, d*V*/d*t* mass and the increase of LVVs, LVIDs (all *P* < .05 D vs C). The significant increase of LVM/BW assessed by echocardiography also manifests the significant development of cardiomyopathy in the 4‐week diabetic rats (*P* < .05 D vs C). The antioxidant treatment with ALP significantly reversed the aforementioned functional changes, preventing left ventricular dysfunction in early diabetes (all *P* < .05 DA vs D).

**Table 2:** M‐mode echocardiographic indices of LV dimension and functions

Parameters	C	D	DA
HR (bpm)	343 ± 10.9	292 ± 2.5*	292 ± 2.8*
LVEF (%)	78.1 ± 3.0	68.2 ± 4.5*	71.8 ± 2.3*
FS (%)	48.9 ± 3.0	40.2 ± 3.8*	42.7 ± 2.0*
LVM (mg)	939.4 ± 16.1	829.2 ± 3.1*	741.8 ± 9.7* ^,^ #
BW (g)	476.2 ± 2.5	340.6 ± 2.7*	343.5 ± 6.3*
LVM/BW	1.97 ± 0.03	2.43 ± 0.02*	2.16 ± 0.06* ^,^ #
LVVd (µL)	342.4 ± 18.8	332.2 ± 15.4	322.5 ± 4.5
LVVS (µL)	97.2 ± 3.3	136.1 ± 9.8*	100.7 ± 13.8#
LVAWd (mm)	2.2 ± 0.08	1.73 ± 0.05*	1.72 ± 0.11*
LVAWs (mm)	3.6 ± 0.13	2.8 ± 0.11*	3.1 ± 0.05* ^,^ #
LVIDd (mm)	7.96 ± 0.19	7.86 ± 0.16	7.76 ± 0.05
LVIDs (mm)	4.1 ± 0.32	5.3 ± 0.16*	4.6 ± 0.28* ^,^ #
LVPWd (mm)	2.1 ± 0.19	1.57 ± 0.09*	1.72 ± 0.15*
LVPWs (mm)	3.3 ± 0.28	2.7 ± 0.10*	2.7 ± 0.04*

Note

All values are means ± SEM, n = 6 per group.

Abbreviations: FS, fractional shortening; HR, heart rate; LV, left ventricle; LVAWd, LV end‐diastolic anterior wall diameter; LVAWs, LV end‐systolic anterior wall diameter; LVEF, LV ejection fraction; LVIDd, LV internal diastolic diameter; LVIDs, LV internal systolic diameter; LVM, LV mass; LVPWd, LV end‐diastolic posterior wall diameter; LVPWs, LV end‐systolic posterior wall diameter; LVVd, LV end‐diastolic volume; LVVs, LV end‐systolic volume.

* *P* < .05 vs C.

^#^ *P* < .05 vs D.

**Table 3:** Haemodynamic variables and indices of cardiac systolic and diastolic function by a pressure‐volume conductance catheter system

Parameters	C	D	D + A
Heart rate (beats/min)	400 ± 17.96	335 ± 19.62*	386 ± 10.43#
Stroke volume (μL)	213.58 ± 33.39	156.58 ± 15.02*	175.18 ± 24.03*
Stroke work (mm Hg/μL)	20 100 ± 1703	10 047 ± 1143*	18 533 ± 2772#
Cardiac output (mL/min)	72.10 ± 1.93	46.97 ± 1.62*	63.17 ± 5.49* ^,^ #
End‐systolic pressure (mm Hg)	133.9 ± 9.64	89.64 ± 4.72*	111.21 ± 6.86* ^,^ #
End‐diastolic pressure (mm Hg)	5.72 ± 1.37	6.06 ± 1.29	2.34 ± 0.26* ^,^ #
Ejection fraction (%)	72.50 ± 11.54	51.45 ± 2.24*	60.44 ± 8.81
Arterial elastance (mm Hg/μL)	0.80 ± 0.10	0.63 ± 0.09*	0.79 ± 0.10#
Maximal slope of systolic pressure increment (mm Hg/s)	9310 ± 718	6893 ± 571*	7688 ± 356*
Diastolic decrement (mm Hg/s)	8907 ± 736	6698 ± 170*	7472 ± 507*
Tau (ms)	10.6 ± 1.75	10.9 ± 1.42	9.5 ± 0.96

Note

All values are expressed as mean ± SEM. n = 6 per group. All of these indices were measured among groups.

* *P* < .05 vs C.

^#^ *P* < .05 vs D.

### 3.3 ALP attenuated oxidative stress and apoptosis induced by hyperglycaemia

**Figure 1:**
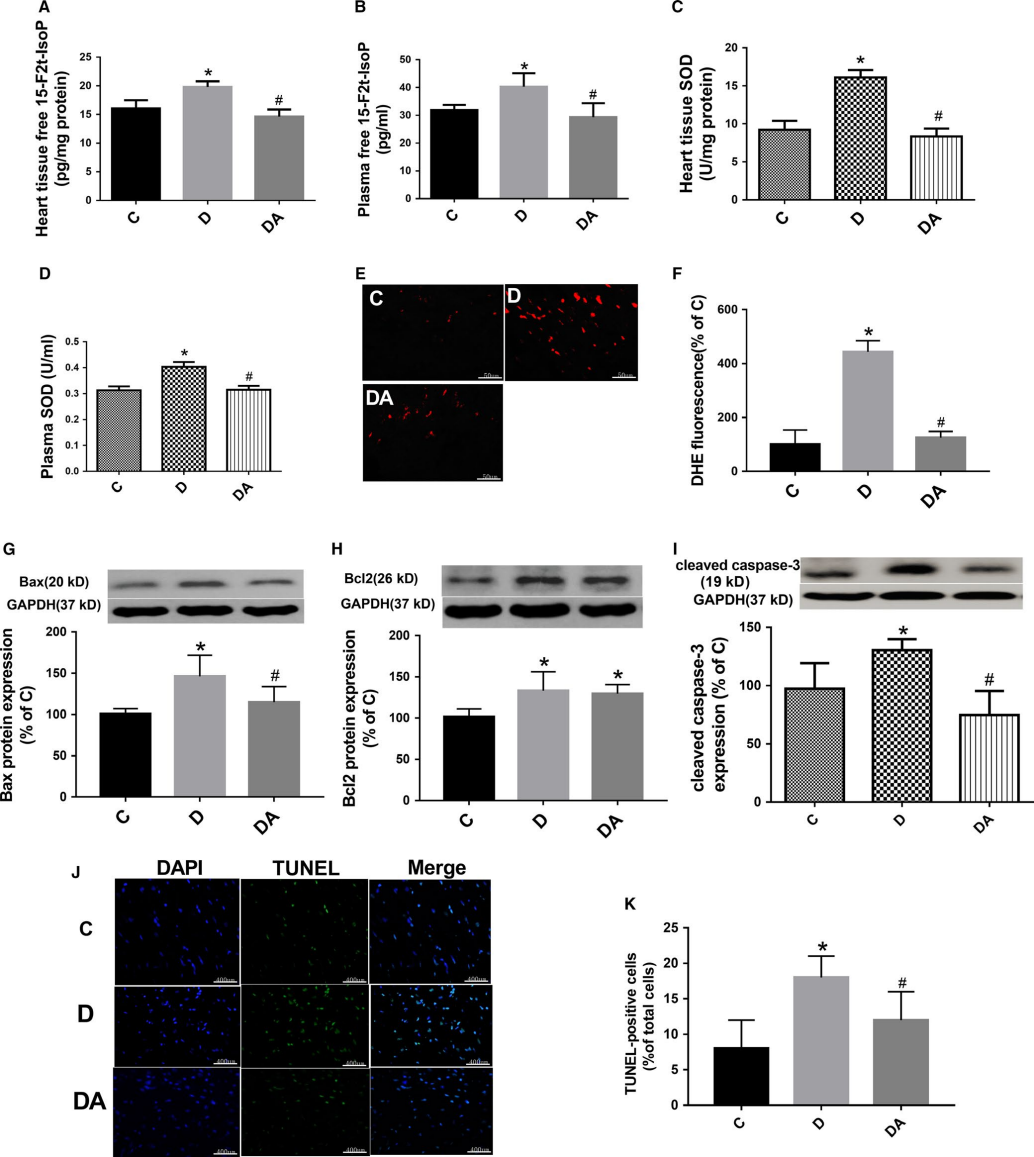
Allopurinol reduced myocardial oxidative stress and apoptosis cell death in 5‐wk streptozotocin‐induced diabetic rats. A, B, Free 15‐F2t‐isoprostane (15‐F2t‐IsoP) in heart tissue was measured by suing an enzyme‐linked immunoassay kit. C, D, SOD activity was detected in cardiac tissue homogenates and plasma, respectively, using commercially available kits (Cayman Chemical). E, F, Myocardial O_2_‐ production assessed by dihydroethidium (DHE) staining (stained in red) and quantification in (C, D) and (DA). G‐I, Protein expressions of cardiac Bax, Bcl2 and cleaved caspase 3. J, K, Myocardial cell apoptosis assessed by terminal deoxynucleotidyl transferase dUTP nick‐end labelling (TUNEL). Data are shown as means ± SEM, with n = 6 animals per group. *P < .05 vs C; ^#^ *P* < .05 vs DA

Both the plasma and cardiac 15‐F2t‐isoprostane (15‐F2t‐IsoP) concentration was significantly higher in diabetic rats than in controls, and ALP normalized the 15‐F2t‐IsoP level in diabetic rats (Figure 1A,B). Similarly, the SOD activity in the plasma and heart tissue significantly increased in diabetic group compared with control group, and the ALP significantly reduced its levels comparable to controls (Figure 1C,D). DHE staining of heart tissue sections showed that superoxide anion (ROS) production (number of DHE‐labelled nuclei, Figure 1E,F) was significantly increased in D rats compared with C rats, and ALP prevented this change in diabetes. Simultaneously, the cardiac proteins expression of Bax and cleaved caspase 3 in diabetes significantly increased compared with controls, and apoptotic cell death was significantly increased despite of moderate and significant increase of cardiac Bcl‐2in the diabetic heart (all *P* < .05 D vs C), and ALP treatment effectively reversed all these changes except that it did not significantly affect the increases in cardiac Bcl‐2 expression in diabetes (all *P* < .05 DA vs D) (Figure 1G‐J).

### 3.4 The feed‐forward loop linking Nrf2, Keap1 and P62 was broken in diabetes and ALP restored it

**Figure 2:**
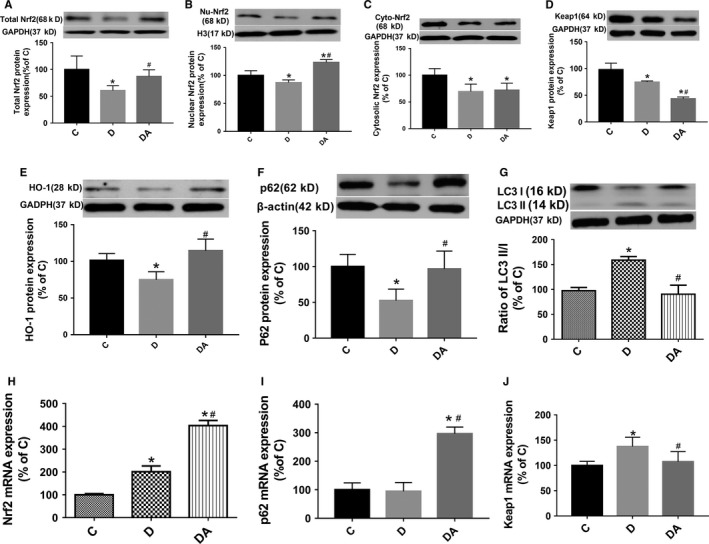
Allopurinol increased the cardiac expression and nuclear translocation of Nrf2 and its down ARE (HO‐1, p62). A‐C, Cardiac expression of total, nuclear and cytosolic Nrf2. D, Cardiac Keap1 protein expression. E, F, Cardiac HO‐1 and p62 protein expression. G, The ratio of LC3 II/LC3 I in the myocardium. H‐J, Cardiac mRNA expression of Nrf2, Keap1 and p62, respectively, calculated against the housekeeping gene GAPDH. Data are shown as mean ± SEM, with n = 6 animals per group. *P < .05 vs C; ^#^ *P* < .05 vs DA; H3, histone H3

In the 5‐week STZ‐induced diabetic rats, both the cardiac cytosolic and nuclear Nrf2 significantly decreased compared with controls, but the nuclear Nrf2 did not decrease as obviously as cytosolic Nrf2 (Figure 2A‐C). In the meantime, protein expression of p62 (Figure 2F) and Keap1 (Figure 2D) went down obviously and the cardiac ratio of LC3 II/I (Figure 2G), an indicator of the extent about autophagy, was significantly increased (all *P* < .05 D vs C). The interruption of the feed‐forward loop in diabetic rats directly resulted in impaired antioxidant capacity manifested as significantly reduced HO‐1 protein expression (Figure 2E) and increased ROS production (Figure 1E,F). After 4‐week treatment with ALP in diabetic rats, p62 significantly increased while the ratio of LC3 II/I was normalized to control level and was significantly higher than that in the untreated D groups. Of note, the nuclear Nrf2 expression was significantly higher in the diabetic treated group than that in the untreated D groups even though the Keap1 expression was obviously further decreased after ALP treatment (all *P* < .05 DA vs D). The mRNA levels of Nrf2 (Figure 2H) and Keap1 (Figure 2J) were increased in the hearts of diabetic rats, and ALP further increased Nrf2 expression but reduced Keap1 gene to a level comparable to the control. The cardiac mRNA levels of p62 did not significantly change in diabetic rats as compared to the control (Figure 2I) but ALP treatment significantly increased cardiac p62 mRNA.

### 3.5 ALP attenuated HG‐induced increase of cardiomyocyte size, oxidative stress, apoptosis and cellular injury via Nrf2 signalling pathway

**Figure 3:**
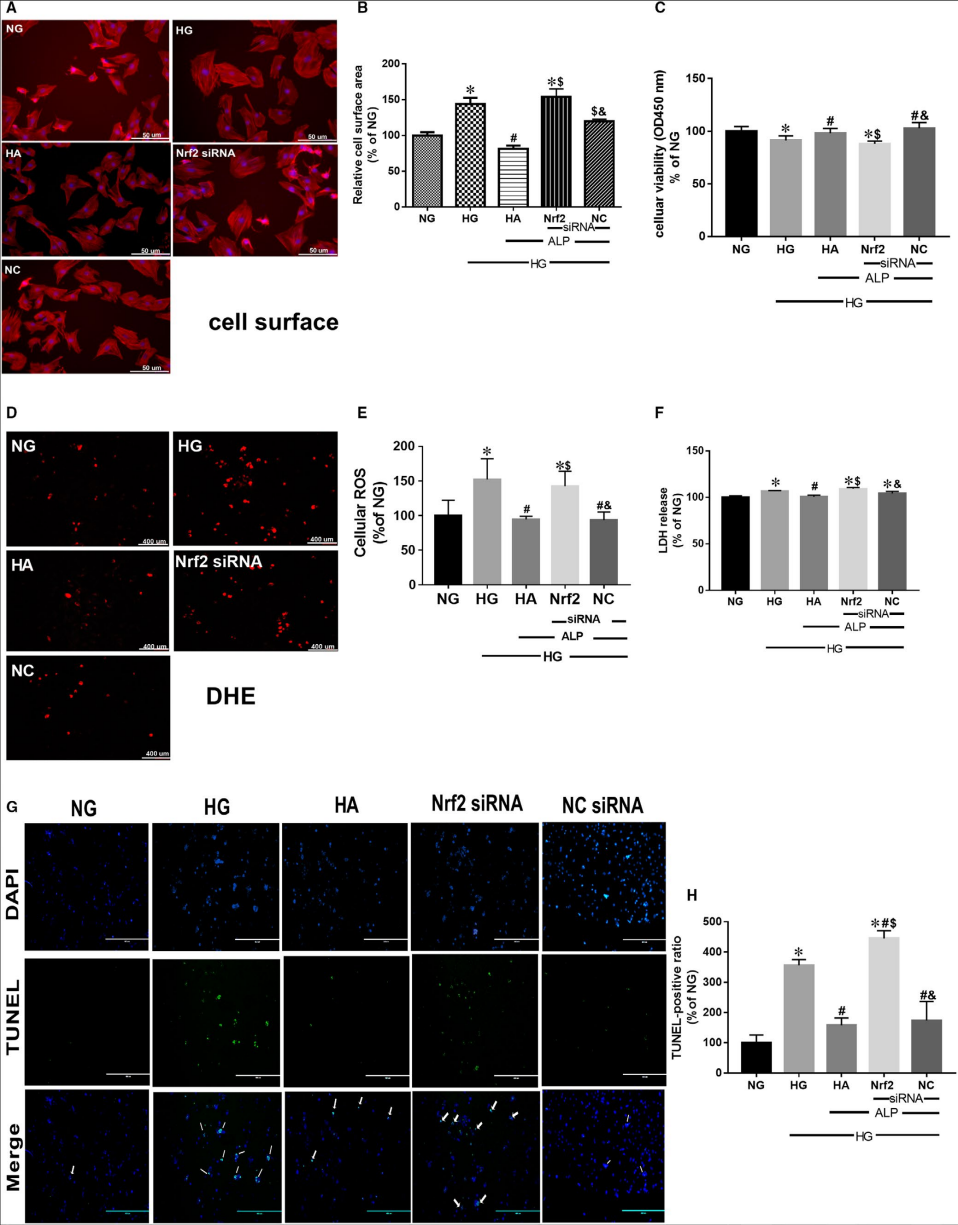
Allopurinol treatment prevented HG‐induced increased cell size and elevated oxidative stress, which can be cancelled by gene silence of Nrf2 in H9C2 cells. H9C2 cardiomyocytes were pretreated with Nrf2 siRNA and control siRNA and then treated with control medium with normal glucose (NG, 5.5 mmol/L glucose) or HG (25 mmol/L glucose) for 48 h; subgroups were treated with ALP for 48 h at the meantime before sample collection. A, B, H9C2 cell size visualization (400 magnification, 50 µm) and quantitation. D, E, Cellular reactive oxygen species production assessed by dihydroethidium (DHE) staining in H9C2 cell (The scale bar in the figure represents 400 µm). C, H9C2 cardiomyocyte viability assessed by CCK8 agent. F, Cardiomyocyte death assessed by lactate dehydrogenase (LDH) release. G, H, Apoptosis of H9C2 myocytes assessed by TUNEL staining (The scale bar in the figure represents 400 µm). Data are mean ± SEM of two independent experiments each performed in triplicate, *P < .05. vs NG; ^#^ *P* < .05 vs HG; ^$^ *P* < .05 vs HA; ^&^ *P* < .05 vs Nrf2

**Figure 4:**
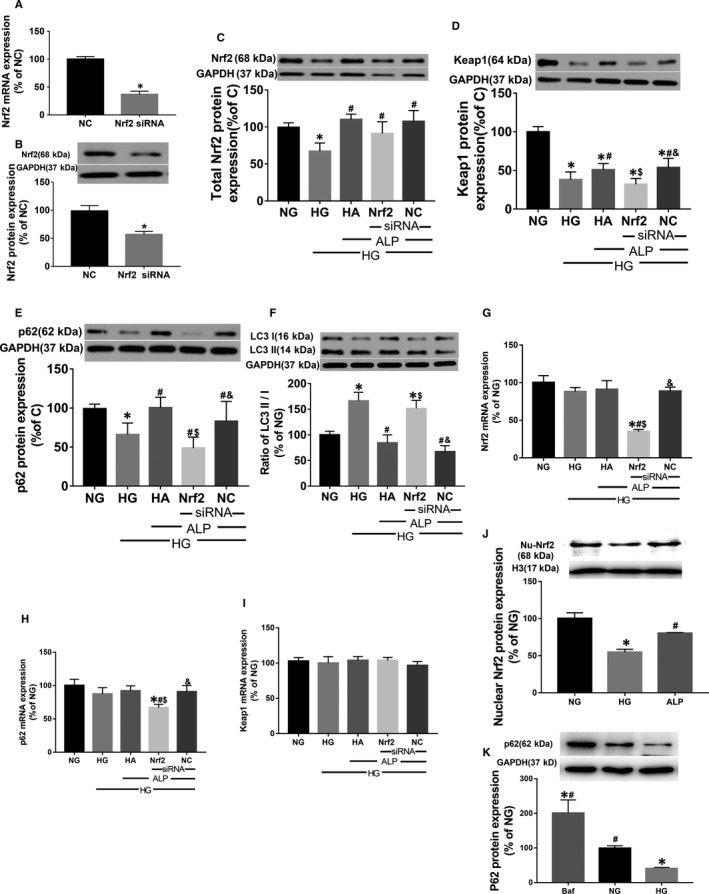
Nrf2/p62 signalling pathway played the key role in the ALP protection to high glucose‐induced cardiomyopathy. A, B, Protein and mRNA expression of Nrf2 after siRNA treatment in the normal glucose. C, Protein expression of Nrf2. D, Protein expression of Keap1. E, Protein expression of p62. F, The ratio of LC3 II/LC3 I in the H9C2 cells. G‐I, The mRNA expressions of Nrf2, p62 and Keap1, respectively, in H9C2 cells, calculated against the housekeeping gene GAPDH. J, Nuclear Nrf2 expression in high glucose cultured H9C2 with or without ALP. K, Treatment with Bafilomycin (Baf), a lysosomal inhibitor that can impair autophagosome‐lysosome fusion and thus track autophagic flux, induced higher P62 protein level than NG group, whereas HG group displayed decreased P62 protein level than NG group. Data are mean ± SEM of two independent experiments each performed in triplicate, *P < .05. vs NG; ^#^ *P* < .05 vs HG; ^$^ *P* < .05 vs HA; ^&^ *P* < .05 vs Nrf2

High glucose significantly increased cell surface area in cultured H9C2 cardiomyocytes (Figure 3A,B) and elevated oxidative stress (Figure 3D,E), concomitantly with increased apoptosis (Figure 3G,H), cellular injury and autophagy manifested as decreased cell viability (Figure 3C) and increased ratio of LC3 II/I (Figure 4F). ALP treatment prevented or reverted these changes. Gene silence of Nrf2 cancelled these protective effects of ALP in H9C2 cells. Furthermore, ALP can up‐regulate the decreased Nrf2 and p62 protein expression induced by high glucose (Figure 4C), whereas it had no impact on the decreased Keap1 (Figure 4D). Nrf2 gene silence did not significantly reduce Nrf2 protein expression in high glucose and ALP treated (HA + Nrf2 siRNA) group despite that it significantly reduced Nrf2 mRNA expression in HA + Nrf2 siRNA group (Figure 4C,G). This is different from the effects that Nrf2 siRNA significantly reduced the expression of Nrf2 both at the mRNA and at protein levels under normal glucose condition (Figure 4A,B). The mRNA expressions of Nrf2, p62 and Keap1 did not significantly change among the NG, HG and HA groups, while the Nrf2 and p62 mRNA significantly decreased in Nrf2 siRNA treated groups (Figure 4G‐I). In vitro, HG caused significant reduction of nuclear of Nrf2, which was reversed by ALP (Figure 4J). However, the protein expression of downstream targeted protein Keap1 and p62 decreased extraordinarily (Figure 4D,E). Besides, Bafilomycin treatment induced higher P62 protein level than NG group (Baf vs NG, *P* < .01), indicating that P62 was accumulated when autophagy was inhibited. Whereas P62 protein levels in the HG group was reduced relative to that in the NG group (HG vs NG, *P* < .05), indicating the excessive autophagy flux in cardiomyocytes under HG condition (Figure 4K).

## 4 DISCUSSION

In the current study, we have demonstrated that antioxidant ALP effectively maintains inner redox homeostasis and attenuates DCM via restoration of Nrf2/p62 signalling pathway through normalizing disordered autophagy and decreasing apoptosis in diabetic rats. We showed that reductions in cardiac nuclear translocation of Nrf2 and in HO‐1 and p62 expressions were accompanied by up‐regulated autophagy, increased apoptosis and cardiac dysfunction in STZ‐induced diabetic rats, which could be reversed by ALP. We further showed that in cultured H9C2 cardiomyocytes, high glucose exposure decreased Nrf2 and p62 protein expression and increased autophagy with concomitant increases in cell size, superoxide anion generation and apoptosis. Similar to that seen in in vivo studies, these adverse changes induced by high glucose in cells could be prevented by ALP administration. However, the protective effects of ALP in cells were cancelled by Nrf2 gene silence. Intriguingly, under high glucose and ALP treatment, Nrf2 protein expression just slightly declined in the cells treated with Nrf2 siRNA, while both the mRNA expression and protein expression of Nrf2 decreased significantly in H9C2 cells receiving Nrf2 siRNA in normal glucose medium. This suggests that there exists other signalling pathway that links ALP to Nrf2. It is noted that ALP enhanced the protein levels of Nrf2 but not that of the mRNA. Thus, possibility exists that ALP may have enhanced Nrf2 protein expression via post‐transcriptional mechanisms that deserve further study. To our best knowledge, this is the first study exploring the role of Nrf2 in antioxidant ALP mediated protection against DCM and cardiac dysfunction.

Excessive oxidative stress induced by hyperglycaemia is a major mechanism of DCM and the subsequent cardiac dysfunction both in human and in animal models of diabetes.^29^, ^36^ In our study, diabetic rats showed asymptomatic cardiac dysfunction manifested as significant reductions in heart rate (HR), left ventricular eject fraction (LVEF), stroke work (SW) and cardiac output (CO), left ventricular end‐systolic volume (LVVs) as compared to non‐diabetic control and ALP improved or normalized HR, LVEF, SW, CO and LVVs in diabetic rats. Biochemically, hearts of diabetic rats displayed excessive oxidative stress manifested as increased levels of 15‐F_2t_‐Isoprostane, increased apoptotic cell death and cardiomyocyte autophagy that were concomitant with decreased HO‐1 expression and reduced superoxide dismutase activity. ALP reverted all the above‐mentioned diabetic‐induced biochemical changes. Results of the current study confirmed the findings of Gao et al^37^ who showed that ALP attenuated left ventricular dysfunction in STZ‐induced diabetic rats and extended to address the mechanism whereby ALP attenuated DCM.

The hyperglycaemia is a key factor that causes mitochondrial superoxide overproduction and oxidative stress, which damages vascular endothelial cells and internal organs in diabetes, particularly the organs with abundant capillary vessels, such as the heart.^38^ Oxidative stress induced by hyperglycaemia has been considered as a major pathogenic cause of DCM^39^ and antioxidant treatment has been proven to be effective in attenuating DCM,^37^, ^40^ as also demonstrated by our study results. We showed that the STZ‐induced diabetic rats developed cardiac dysfunction, concomitantly with aggravation of oxidative stress (increased 15‐F2t‐Isoprostane and ROS) while antioxidant ALP can reduce hyperglycaemia‐induced ROS and oxidative stress and attenuate cardiac dysfunction. ALP has been shown to reduce both the formation of uric acid and the production of oxidative free radicals^41^ by restoring Nrf2 and HO‐1 expressions,^42^ and enhancement of Nrf2 has recently been shown to be a mechanism by which treatments attenuate DCM.^43^, ^44^ We thus postulated that enhancement of Nrf2 may be a major mechanism whereby ALP attenuates DCM. This mechanism may also explain why ALP could attenuated DCM despite it had no significant effect on plasma levels of glucose in diabetes.

There is general consensus that low levels of ROS act as intracellular signal transducers that activate autophagy, promoting cell survival, whereas high levels of ROS induce apoptosis by damaging cellular components. It is interesting to note that the redox system can potentially be a switch of autophagy and apoptosis and function as a crosstalk in between autophagy and apoptosis.^45^ Coincidentally, our results showed increases in autophagy and apoptosis simultaneously occurred in the condition of high ROS both in vivo and in vitro. ALP reduced apoptosis and attenuated autophagy in diabetic or high glucose condition. Findings of our current study provided a hint that Nrf2 pathway might regulate the crosstalk in between apoptosis and autophagy in the diabetic myocardium. It is worth noting that ALP treatment did not improve the Bcl2 levels in diabetic rats, which means that other pathways linking apoptosis and autophagy may also play a role in the protective effect of ALP. Transactivation of antioxidant genes may block apoptosis and serve as a feedback loop to reduce autophagy. In addition to oxidative stress, nutrient deprivation resulted from metabolic disorder of glucose also activates autophagy, which acts to restore metabolic homeostasis via the degradation of macromolecules to provide nutrients.^46^ In the present study, antioxidant treatment with ALP can effectively attenuate DCM through reducing apoptosis, normalizing autophagy level and further activate Nrf2 as compared to diabetic rats.

Nrf2, a vital cellular redox‐sensitive transcription factor, responses to oxidative stress and regulates its downstream antioxidant defence mechanisms, maintaining the cellular redox homeostasis. In this study, cardiac Nrf2 expression significantly decreased in the nucleus both in 4‐week diabetic rats and in H9C2 cells treated with high glucose, concomitant with reduced expression of HO‐1, p62 and Keap1, an inhibitor protein of Nrf2. The variation of diabetic cardiac Nrf2 protein expression in our current study is in line with findings of our previous study^10^ and others.^47^ Nrf2 has been widely accepted as a protective factor against oxidative stress, and cardiac Nrf2 expression has been shown to be reduced both in diabetic animals and in patients.^48^ Diabetes may up‐regulate antioxidants in the heart as compensative mechanisms in the early phase, and dysregulation of autophagy was shown to result in prolonged Nrf2 activation in a p62‐dependent manner.^49^ p62, an autophagy adaptor and acceptor, has a Keap1‐interacting region (KIR) domain, which allows p62 to sequester Keap1 into the autophagosomes, impairing the ubiquitylation of Nrf2 and thus leading to further activation of the Nrf2 signalling pathway.^13^ In the present study, the ratio of cardiac LC3 II/I significantly increased in diabetic rats that was concomitant with decreased p62 and Keap1 protein expression but also reduced Nrf2 protein expression as compared to the control. This indicates that activated autophagy can degrade more p62‐Keap1 complexes and subsequently affect nuclear translocation of Nrf2 in 4‐week diabetic rats and in H9C2 cells treated with high glucose for 48 hours. Excessive ROS should be responsible for this broken Nrf2/p62 pathway. In normal state, the loop of Nrf2‐Keap1‐P62 is in the condition of a dynamic steady status, maintaining cellular redox homeostasis. Once in the status of diabetes, disrupted internal environment produce excessive ROS and the consequently launched autophagy, which prompts p62 to bind with Keap1 and subsequently inactivates Nrf2, causing a series of consequent complications, such as cardiac dysfunction. AP‐mediated reduction of ROS generation and cardioprotective effects should be largely attributable to Nrf2 activation rather than just targeting xanthine oxidase enzyme, which were evidenced by the findings that ablation of Nrf2 expression in vitro cancelled all the protective effects of AP on high glucose‐treated H9C2 cells. However, whether or not there exists an upstream or downstream regulation between xanthine oxidase inhibition and Nrf2 activation needs to be further explored.

In recent years, antioxidant therapy has been extensively used to counteract excessive ROS in diabetes. However, controversies exit that question the beneficial effects of antioxidant therapy.^50^ This is largely because that the mechanism of antioxidant therapy is complicated. Of note, the cardioprotection of ALP was obviously observed in diabetic rats in this study, as well as in Szwejkowski's study.^51^ In addition to the functional studies in vivo using both intra cardiac and echocardiographic approaches, we also detected the signalling changes that involve the aforementioned Nrf2 pathway in vivo and in vitro. After 4 weeks treatment with ALP in diabetic rats, the ratio of LC3 II/I reduced and P62 protein expression increased to levels comparable to that in the control group, while lower protein expression of Keap1 was accompanied with higher protein expression of Nrf2 in the nucleus as compared to untreated diabetic group. In vitro, Nrf2 gene silence cancelled the cardiac protective effects of ALP against high glucose‐induced H9C2 injuries. Thus, it is plausible that ALP confers cardioprotection in diabetes by normalizing the disordered autophagy and to consequently restore the disrupted Nrf2‐Keap1‐p62 loop in diabetes.

Diabetic cardiomyopathy occurred in patients with both type 2 and type 1 diabetes, and the mechanisms and clinical features of diabetic cardiomyopathy may be partially distinct in type 1 vs type 2 diabetes.^52^ With the development of diabetes, hyperglycaemia‐induced oxidative stress and damage have been considered as a major mechanism of diabetic cardiomyopathy.^53^ In the current study, we confirmed the development of diabetic cardiomyopathy STZ‐induced type 1 diabetic rats by evaluating cardiac function, pathological changes and biological abnormality and demonstrated the effectiveness and mechanism of the antioxidant ALP on DCM.

## 5 CONCLUSION

**Figure 5:**
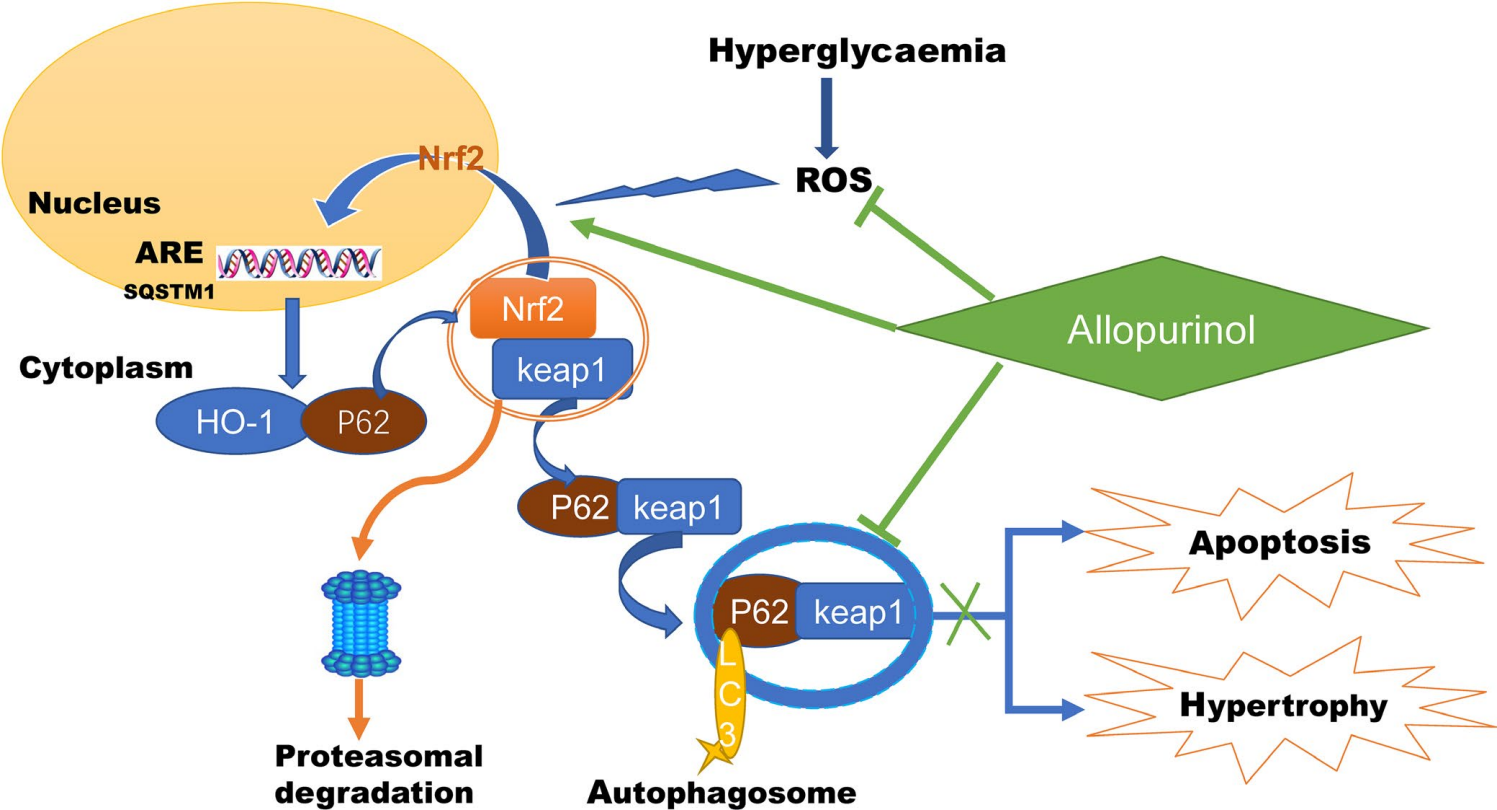
Schematic of proposed signalling involved in ALP attenuates DCM and cardiac dysfunction via activating Nrf2 pathway. Hyperglycaemia‐induced oxidative stress destructs Nrf2 signalling by inhibiting its nuclear translocation, which concomitantly with activated autophagy and subsequently reduced HO‐1 and p62 expression, resulting in cardiomyocytes apoptosis and cardiac hypertrophy. ALP activates Nrf2 by increasing its nuclear translocation following with target on ARE elements (including p62 and HO‐1) and concomitantly normalizes autophagy, which in turns reduces myocardial oxidative stress, attenuates cardiac hypertrophy and apoptosis and eventually improves cardiac function

The findings of the current study suggest that the impaired feed‐forward loop linking Nrf2, p62 and autophagy is a critical mechanism for the out‐of‐balance redox homeostasis induced by hyperglycaemia in type 1 diabetes as illustrated (Figure 5), which eventually result in the development of DCM. In the early stage of diabetic rats, diabetes exited some scathing heart functional changes that can be detected by the sensitive echocardiography and PV‐loop system, including the lower HR, LVEF, stroke work and cardiac output. The excessive oxidative stress is the main pathogenesis for the broken of the feed‐forward loop and subsequent recession of antioxidant capability. Thus, restoration of the disorganized autophagy and the subsequent repairing of Nrf2/p62 pathway may represent a major mechanism whereby ALP confers its cardioprotection against DCM. Nrf2 is the key director in this scenario.

## CONFLICT OF INTEREST

The authors confirm that there are no conflicts of interest.

## AUTHOR CONTRIBUTIONS

J. Luo and Z. Xia designed the study. J. Luo, D. Yan, S. Li, F. Zeng and S. Liu conducted the experiments. J. Luo, C. W. Cheung, M. G. Irwin and Z. Xia analysed the data. J. Luo and D. Yan wrote the manuscript. Z. Xia and H. Huang revised the manuscript. All authors read the final version of the manuscript. All authors approve and agree to be responsible for all aspects of this work.

## Data Availability

The data are available from the corresponding authors upon reasonable request.
